# Recombinant human insulin-like growth factor-1 promotes osteoclast formation and accelerates orthodontic tooth movement in rats

**DOI:** 10.1590/1678-7757-2020-0791

**Published:** 2021-05-14

**Authors:** Ju-Xiang Peng, Xiao-Yan Guan, Gao-Hua Li, Jian-Li Zhong, Ju-Kun Song, Lin-Lin Xiao, Su-Han Jin, Jian-Guo Liu

**Affiliations:** 1 Guiyang Stomatological Hospital Affiliated to Zunyi Medical University Guiyang Hospital of Stomatology Department of Orthodontic Guiyang China Guiyang Stomatological Hospital Affiliated to Zunyi Medical University, Guiyang Hospital of Stomatology, Department of Orthodontic, Guiyang, China.; 2 Zunyi Medical University School of Stomatology Department of Orthodontic Zunyi China Zunyi Medical University, School of Stomatology, Department of Orthodontic, Zunyi, China.; 3 Shenzhen Ai Kang Jian Stomatological Hospital Outpatient Department of Stomatology Shenzhen China Shenzhen Ai Kang Jian Stomatological Hospital, Outpatient Department of Stomatology, Shenzhen, China.; 4 Guangdong Province Stomatological Hospital Department of Orthodontic Guangzhou China Guangdong Province Stomatological Hospital, Department of Orthodontic, Guangzhou, China.; 5 Guizhou Province People’s Hospital Department of Oral and Maxillofacial Surgery Guiyang China Guizhou Province People’s Hospital, Department of Oral and Maxillofacial Surgery, Guiyang, China.; 6 Special Key Laboratory of Oral Diseases Research from Higher Education Institution of Guizhou Province & Zunyi Key Laboratory of Oral Disease Research Zunyi China Special Key Laboratory of Oral Diseases Research from Higher Education Institution of Guizhou Province & Zunyi Key Laboratory of Oral Disease Research, Zunyi, China.

**Keywords:** Insulin-like growth factor I, Osteoclasts, Orthodontics, Tooth movement techniques

## Abstract

Background: IGF-1 may be an important factor in bone remodeling, but its mechanism of action on osteoclasts during orthodontic tooth movement is complex and unclear. Methodology: The closed-coil spring was placed between the left maxillary first molar and upper incisors with a force of 50 g to establish an orthodontic movement model. Eighty SD rats were randomized to receive phosphate buffer saline or 400 ng rhIGF-1 in the lateral buccal mucosa of the left maxillary first molar every two days. Tissue sections were stained for tartrate-resistant acidic phosphatase (TRAP), the number of TRAP-positive cells was estimated and tooth movement measured. Results: The rhIGF-1 group exhibited evidential bone resorption and lacuna appeared on the alveolar bone compared to the control group. Moreover, the number of osteoclasts in compression side of the periodontal ligament in the rhIGF-1 group peaked at day 4 (11.37±0.95 compared to 5.28±0.47 in the control group) after the orthodontic force was applied and was significantly higher than that of the control group (p<0.01). Furthermore, the distance of tooth movement in the rhIGF-1 group was significantly larger than that of the control group from day 4 to day 14 (p<0.01), suggesting that rhIGF-1 accelerated orthodontic tooth movement. Conclusion: Our study has showed that rhIGF-1 could stimulate the formation of osteoclasts in the periodontal ligament, and accelerate bone remodeling and orthodontic tooth movement.

## Introduction

Currently, the research on immune biomarkers of tooth root surrounding tissues is a hot spot.^1^^–^^3^ Orthodontic tooth movement depends on the remodeling of tissues surrounding the roots. The periodontium is composed of three closely related structures: alveolar bone, periodontal ligament (PDL) and cementum, which plays an important role in orthodontic tooth movement^4^. The periodontal ligament connects the cementum to the alveolar bone by Sharpey’s fibers and undergoes remodeling in homeostasis and orthodontic tooth movement. Osteoclasts derive from the hematopoietic/monocyte lineage and are replaced every few months in the periodontal ligament and alveolar bone.^5^

Accelerating orthodontic tooth movement could shorten treatment duration and in combination with corticectomy-assisted tooth movement could promote post-treatment stability and has been intensively investigated.^6^ The remodeling of the periodontal tissue relies on the physiological process of balancing osteoblasts and osteoclasts under the regulation of various biological factors and complicated molecular mechanisms to lead to tooth movement due to mechanical force. Hu, et al.^7^ (2016) investigated the effects of recombinant growth hormone on orthodontic tooth movement in rats and found that recombinant growth hormone treatment increased the number of insulin-like growth factor-1 (IGF-1) positive osteoclasts in the periodontal ligament and accelerated tooth movement.^7^ Xu, et al.^8^ (2014) showed that local injection in rats of recombinant human TGF-α1 noticeably increased the number of osteoclasts and stimulated tooth movement.

IGF-1 is crucial for bone cell function and skeletal development and maintenance and is the major mediator of growth hormone-induced bone growth. Osteocytes express high quantities of IGF-1,^9^ which is one of the earliest bone responses to mechanical loading.^10^ IGF-1 may be an important factor in bone remodeling, and changes in IGF-1 content during tooth movement in orthodontic patients may be involved in alveolar bone remodeling.^11^ The mechanical load of PDL is closely related to the autocrine/paracrine expression of IGF components, which leads to a long-term organized remodeling of alveolar bone.^12^ IGF-1 may also modulate the bone remodeling process by its actions on osteoblasts and osteoclasts. Osteoclasts are the major cell for bone resorption, which plays a pivotal role in remodeling the alveolar bone during the movement of orthodontic tooth.^13^^,^^14^ The study showed that recombinant human growth hormone could stimulate IGF-1 expression in PDL, and accelerate bone remodeling and tooth movement.^7^ Recombinant human IGF-1 (rhIGF-1) is now in the research spotlight because of the role of IGF-1 in tooth bone remodeling and movement. Our study aimed at investigating the effect of rhIGF-1 on the number of osteoclast formation in periodontal tissue and changes of orthodontic tooth movement in SD rats.

## Methodology

### Animals

The study protocol was approved by the local ethics committee at the authors’ affiliated hospital. Animal study was conducted in strict accordance with the established institutional guidelines on the use of experimental animals.

**Figure 1:**
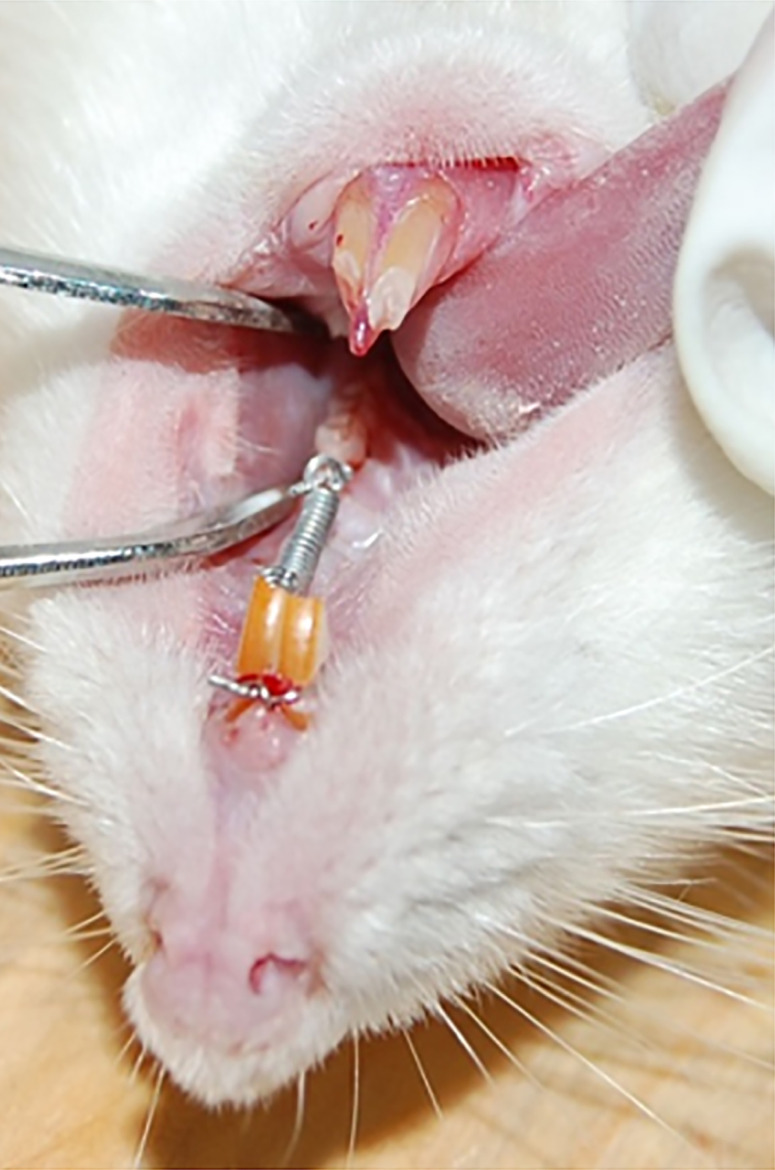
The orthodontics appliance and rat tooth movement model. The Ni-Ti closed-coil spring was wrapped with orthodontic ligation wire, a 50 g force was applied to the left first molar of the rats, and the other end was attached to the ipsilateral incisor

Eighty 6-8 weeks old male Sprague-Dawley (SD) rats (Animal permit No.: SCXK (Yu) 2007017; the Experimental Animal Center of the Third Military Medical University, Chongqing, China), weighing 200-250 g each, were housed at a constant temperature (20-22°C) at 50-70% humidity with a 12 h light/dark photoperiod. They were allowed one week to accommodate. Then, the rats were placed in the supine position and received an intraperitoneal injection of 10% chloral hydrate (0.35 mL/100 g). The fine emery of turbine was used to grind 0.2 mm grooves mm on the medial buccal and lateral axis of the tongue on the gingival of the left maxillary first molar and the upper maxillary incisor for fixation of the wire ligation. The two maxillary incisors acted as the anchorage teeth, with a designed force of 50 g with the use of GAC Ni-Ti spiral spring (Shanghai, China) between the upper incisor and the first molar to move the left maxillary first molar mesially.^7^^,^^15^ Moreover, the lower anterior teeth were ground to prevent the breakage of appliance (Figure 1). The appliances were monitored regularly in case there was any breakdown. All rats were fed with softer foods on the first two days after the appliances were used.

The rats were then randomized to receive phosphate buffer saline (PBS) (the control group) or 400 ng rhIGF-1 (Pepro-Tech, USA) (the rhIGF-1 group) in the lateral buccal mucosa of the left maxillary first molar every two days.^16^

### TRAP staining

The rats were sacrificed by transcranial perfusion at days 1, 4, 7, 10, and 14 after the orthodontic force was applied, and subjected to fixation with 4% paraformaldehyde. The left ventricle was thoroughly rinsed with 250 mL normal saline at 37°C. The left maxillary was preserved in 4% paraformaldehyde for 18 to 24 hours at 4°C and then decalcified at 4°C for up to a month and a half in 15% ethylenediaminetetraacetic acid (EDTA, pH 7.4), which was replenished every week until the preserved sample was penetrated easily with a pin. The palatal side of the sample was trimmed and rendered parallel with the main axis of the first molar. The specimens underwent gradient alcohol dehydration and xylene replacement, followed by paraffin embedding. The molar was sectioned in the sagittal plane at a thickness of 4 μm, and then mounted on a polylysine processed glass slide.

Staining for tartrate-resistant acidic phosphatase (TRAP), a marker enzyme for odontoclasts, was performed with the TRAP assay kit following the manufacturer’s recommendations (Sigma Aldrich, St. Louis, MO, USA) and TRAP-positive cells were estimated by examining two sections per sample. Odontoclasts in four randomly chosen visual fields (×400) covering more than one-third of the periodontal membrane and the area of the alveolar bone on the compression side of the root apex were estimated. The staining intensity was evaluated with the use of Image-Pro Plus 6.0 (USA). In our study, Odontoclasts represented the multi-core eosinophilic giant cells on the surface of the alveolar bone or the lacunae of broken bone on the edge of irregular bones.

### Measurement of tooth movement

A single tray of potassium alginate impression material was used to take the maxillary impression of rats before and after the pressure side dental operation, and the impression was perfused to form a plaster model.^17^ The models were placed horizontally on the observation platform of the XTL-3400C stereomicroscope (Shanghai, China). The conjunctive plane was parallel to the ground, and was of the same height as the observation platform. Images were captured using a JVC digital camera with 40× magnification to set the length scale. The model image was acquired using a computer, and the proximal middle lingual groove of the first molar and the distal middle surface of the second molar were marked. The distance between the two points was determined at least three times independently using YR-MV1.0 microscopic image measurement software^18^ and the mean value was used. The distance difference between two points before and after the operation is the distance of the first molar on the afterburner side.

### Statistical analysis

Data were presented as mean ± standard deviation (*x̅*±sd). Statistical analysis was performed with the SPSS version 16.0 software (SPSS Inc., Chicago, IL, USA). A one-way analysis of variance (ANOVA) was applied for comparison between the two groups for the number of osteoclasts at different time points of compression. The *t*-test was applied for the comparisons of tooth movement and the number of osteoclasts between two groups. A p<0.05 represented the significant statistical difference.

## Results

### rhIGF-1 promotes the osteoclasts formation on the compression side of the alveolar bone surface

**Figure 2:**
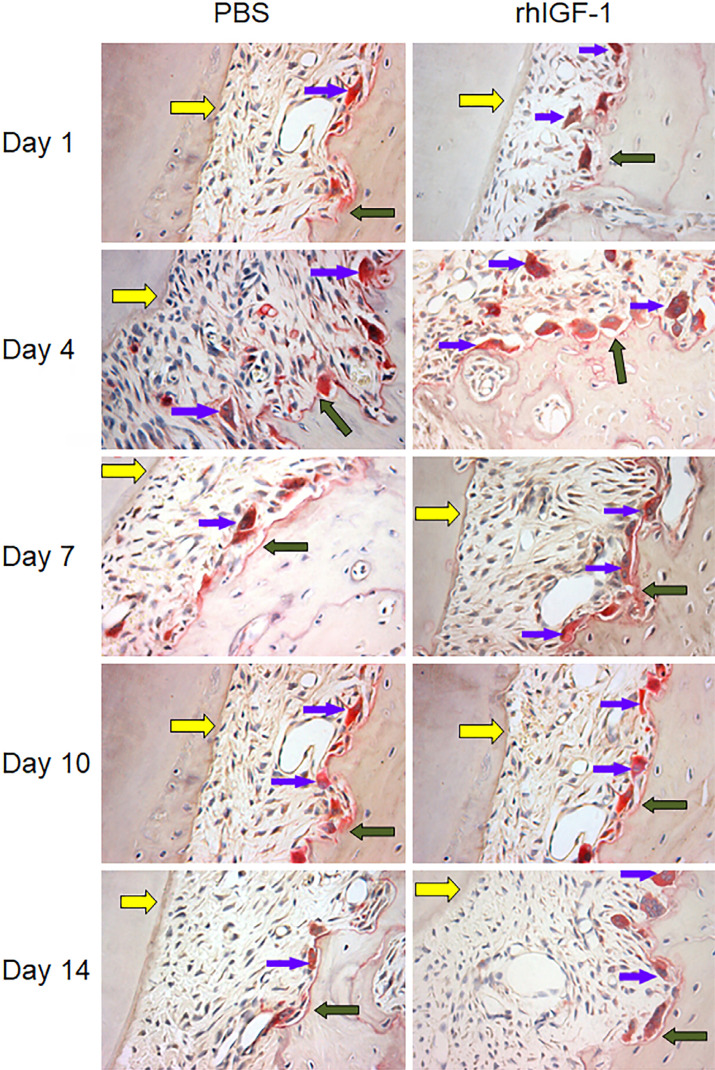
Histological observations of osteoclasts on the compression side. The red areas shown by the blue arrow are osteoclasts, the blue arrows indicate osteoclasts in red stained for TRAP, the yellow arrows indicate the cementum, and the green arrows indicate the alveolar bone. Magnification, ×400

Figure 2 shows the distribution of pressure-side osteoclasts in the rhIGF-1 group and the control group at different time. Histological observations showed few osteoclasts, which had a small cell volume and less abundant nucleus, at Day 1 after the orthodontic force was applied in both groups. At Day 4, the number and volume of osteoclasts on the pressure side increased, and obvious bone resorption lacunae appeared on the alveolar bone. At Day 7, the cell volume decreased and the bone lacunae were flat. The number of osteoclasts did not change significantly from Day 10 to Day 14.

**Figure 3:**
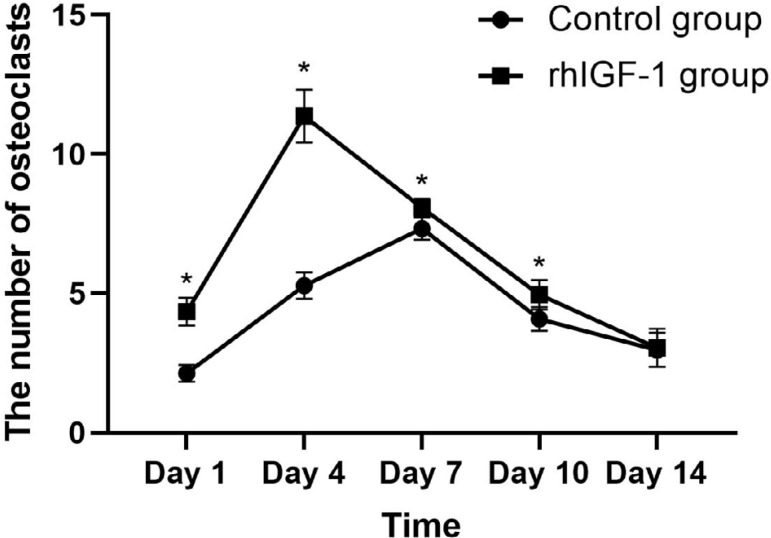
Changes of the number of osteoclasts in periodontal ligament of rats in two groups from 1 to 14 days after orthodontic force was applied. *: p<0.01, compared to the control group

Moreover, the number of osteoclasts in compression side of the periodontal ligament in the rhIGF-1 group peaked at day 4 (11.37±0.95, compared to 5.28±0.47 in the control group) after the orthodontic force was applied, and was significantly higher than that of the control group at day 1, 4, 7 and 10 (p<0.01) (Figure 3). Afterwards, the number of TRAP-positive cells decreased in the rhIGF-1 group and remained stable. However, the activation and accumulation of TRAP-positive cells were observed at day 7 in the control group.

### rhIGF-1 accelerates orthodontic tooth movement

We determined orthodontic tooth movement at different time points. Both groups showed time-dependent increase in the distance of orthodontic tooth movement (Table 1). Furthermore, the distance of tooth movement in the rhIGF-1 group was significantly larger than that of the control group from Day 4 to Day 14 (p<0.01), suggesting that rhIGF-1 accelerated orthodontic tooth movement.

**Table 1:** Tooth movement of two groups (mm, mean±sd, n=8)

Time	Control group (A)	rhIGF-1 group (B)
1d	0.040±0.040	0.076±0.045
4d	0.164±0.010	0.222±0.010*
7d	0.267±0.013	0.369±0.007*
10d	0.339±0.015	0.481±0.008*
14d	0.410±0.014	0.581±0.009*

* P<0.01 compared to the control group

## Discussion

Bone regeneration is a complicated process involving multiple cell types and numerous factors, with a finely tuned balance between bone formation and bone resorption mediated by osteoblasts and osteoclasts that are subject to direct regulation by local cytokines. Bone IGF is one of the most abundant cell growth factors in the bone, and plays a pivotal role in the regulation of bone formation and resorption. Our study showed that rhIGF-1 significantly stimulated the formation of osteoclasts in the alveolar bone, promoted bone resorption and enhanced orthodontic tooth movement in rats, suggesting that rhIGF-1 could be a promising therapeutic agent for accelerating orthodontic tooth movement.

Several studies^19^ have shown that IGF-1 influences the activity of both osteoblasts and osteoclasts on the compression side, which promotes bone reconstruction. Reports supported by Kheralla, et al.^12^ (2010) revealed that IGF-1 can induce the transformation of stromal cells and mediate the differentiation of osteoclasts and osteoblasts, with the ability to regulate bone reconstruction. It is a mitogenic factor and promotes cellular proliferation and differentiation. The activation of osteoclasts plays an important role in the reconstruction of alveolar bone. Mochizuki, Sakai and Iwashita^20^ (2006) demonstrated that the number of TRAP-positive cells increased evidently with different doses of IGF-1 in the presence of 1,25-(OH)2D3 and macrophage colony stimulating factor (M-CSF), indicating the function of IGF-1 in promoting the differentiation from osteoclast precursors to osteoclasts. The differentiation of osteoclasts plays an important role in the process of orthodontic tooth movement.^21^ In our study, 400 ng rhGF-1 was administered based on the doses of earlier experiments and noticeably increased the number of osteoclasts on the compression side of the first molar when compared with the control group. At Day 4 after the mechanical force was applied on the orthodontic tooth, numerous TRAP-positive cells were observed on the surface of the alveolar bone, which were activated and accumulated around the bone resorption lacuna, indicating an active bone absorption. The number of osteoclasts peaked earlier in the rhIGF-1 group, which is consistent with greater orthodontic tooth movement when compared with the control group, revealing that rhIGF-1 could promote osteoclast proliferation and differentiation, and accelerate bone reconstruction. Although the selection of random field images in our statistical analysis may have influenced the results, the research of other scholars also supports our results. These results suggested that IGF-1 might participate in alveolar bone reconstruction by stimulating osteoclast differentiation and proliferation partially, and promote orthodontic tooth movement.

Studies^22^^–^^26^ have shown that IGF-1 influences the differentiation and proliferation of osteoclasts. However, the potential mechanism of IGF-1 that promotes the proliferation and differentiation of osteoclasts is complex and unknown. Some studies found that osteoprotegerin (OPG) was associated with tooth movement.^27^^,^^28^ Hill, Reinolds and Meikle^29^ (1995) found that IGF modulated bone absorption by regulating OPG. Kobayashi, et al.^30^ (2000) showed that the expression of RANKL mRNA increased evidently in the maxillary first molar by applying the force on orthodontic tooth. RANKL is an essential factor for osteoclast differentiation, which is expressed on the surface of osteoblasts by binding to osteoclast or the RANK receptor on the surface of its precursor cells to stimulate the formation and differentiation of osteoclasts.^31^^,^^32^ In the process of tooth movement, the OPG/RANK/RANKL system^33^^,^^34^ is the key in regulating osteoclast differentiation and alveolar bone reconstruction. The aforementioned studies have suggested that IGF-1 may indirectly act on osteoclasts through the OPG/RANK/RANKL system and regulate the differentiation and proliferation of osteoclasts. Exogenous rhIGF-1 can promote remodeling of orthodontic periodontium by acting on osteoclasts to accelerate the movement of teeth. However, further studies are needed to confirm the function of IGF-1 and its mechanism in promoting the reconstruction of orthodontic periodontal tissues.

## Conclusion

In conclusion, our study showed that rhIGF-1 could stimulate the formation of osteoclasts in the periodontal ligament and accelerate bone remodeling and orthodontic tooth movement.
